# Draft Genome Sequence of *Salmonella* *enterica* Serovar Typhi IMR_TP298/15, a Strain with Intermediate Susceptibility to Ciprofloxacin, Isolated from a Typhoid Outbreak

**DOI:** 10.1128/genomeA.01740-16

**Published:** 2017-03-02

**Authors:** Norazah Ahmad, Shirley Yi Fen Hii, Rohaidah Hashim, Rahizan Issa

**Affiliations:** Bacteriology Unit, Institute for Medical Research, Kuala Lumpur, Malaysia

## Abstract

*Salmonella enterica* serovar Typhi with reduced susceptibility to ciprofloxacin is increasingly being reported globally. An outbreak caused by *Salmonella* Typhi with decreased ciprofloxacin susceptibility has not been reported before in Malaysia. We present here the annotated draft genome of a *Salmonella* Typhi strain involved in a typhoid outbreak.

## GENOME ANNOUNCEMENT

*Salmonella enterica* serovar Typhi is the causative agent of typhoid fever and remains common in many developing countries. WHO estimates that approximately 21 million cases and 222,000 typhoid-related deaths occur annually worldwide (1). Traditionally, ampicillin, chloramphenicol, and trimethoprim-sulfamethoxazole are used for treatment of typhoid fever. However, resistance to these antimicrobials has been increasingly reported, leading to the increased use of fluoroquinolones such as ciprofloxacin. Ciprofloxacin is currently recommended as the first choice of treatment for adults with typhoid fever in Malaysia (http://www.pharmacy.gov.my/v2/sites/default/files/document-upload/national-antibiotic-guideline-2014-full-versionjun2015_1.pdf). An outbreak caused by Salmonella Typhi strains with decreased ciprofloxacin susceptibility has not been reported before in Malaysia. This strain was isolated from the stool of a patient during an outbreak of typhoid which occurred in October 2015.

DNA of Salmonella Typhi IMR_TP298/15 was extracted using the Masterpure DNA purification kit (Epicentre, Madison, WI, USA). The DNA library was prepared following the Illumina protocol using the Nextera XT DNA sample prep kit (Illumina, San Diego, CA, USA). Whole-genome sequencing was performed using an Illumina Miseq (Illumina, Inc.) system. A total of 1,407,232 filtered reads were generated with an average read length of 225 bp. *De novo* assembly was performed using CLC Genomics Workbench (version 8.5.1; CLC Bio-Qiagen, Aarhus, Denmark) and yielded 172 contigs with a total length of 4,822,759 bp and G+C content of 52.04%. The assembly *N*_50_ was 98,030 bp and the sequence length of the longest scaffold was 289,444 bp. The gene prediction on the assembled scaffold was done using Prodigal version 2.60 (2). Total RNA and rRNA were predicted using ARAGORN version 1.2.34 (3) and RNAmmer version 1.2 (4), respectively. A total of 4,658 open reading frames, 61 tRNAs, and four rRNAs were predicted.

The isolate showed a Ser83 > Phe mutation in the QRDR of the *gyr*A gene. This is one of the common point mutations in the *gyr*A gene that is found to be associated with quinolone resistance (5).

Multilocus sequence typing (MLST) analysis revealed that this strain belongs to sequence type 2 (ST2) (http://mlst.warwick.ac.uk/mlst/dbs/Senterica).

### Accession number(s).

This whole-genome shotgun project has been deposited at DDBJ/EMBL/GenBank under the accession no. LUHL00000000, which is the version described in this paper.
